# Breast magnetic resonance imaging as a problem solving tool in women recalled at biennial screening mammography: A population-based study in the Netherlands

**DOI:** 10.1016/j.breast.2021.11.014

**Published:** 2021-11-20

**Authors:** Jessie JJ. Gommers, Adri C. Voogd, Mireille JM. Broeders, Vivian van Breest Smallenburg, Luc JA. Strobbe, Astrid B. Donkers - van Rossum, Hermen C. van Beek, Ritse M. Mann, Lucien EM. Duijm

**Affiliations:** a Department of Medical Imaging, Radboud University Medical Center, Geert Grooteplein 10, 6525, GA, Nijmegen, the Netherlands; b Department of Epidemiology, Maastricht University Medical Center, Universiteitssingel 60, 6229, ER, Maastricht, the Netherlands; c Department of Research and Development, Netherlands Comprehensive Cancer Organization, Godebaldkwartier 419, 3511, DT, Utrecht, the Netherlands; d Department for Health Evidence, Radboud University Medical Center, Geert Grooteplein 10, 6525, GA, Nijmegen, the Netherlands; e Dutch Expert Center for Screening, Wijchenseweg 101, 6538, SW, Nijmegen, the Netherlands; f Department of Radiology, Jeroen Bosch Hospital, Henri Dunantstraat 1, 5223, GZ, ‘s-Hertogenbosch, the Netherlands; g Department of Surgical Oncology, Canisius Wilhelmina Hospital, Weg Door Jonkerbos 100, 6532, SZ, Nijmegen, the Netherlands; h Department of Radiology, Catharina Hospital, Michelangelolaan 2, 5623, EJ, Eindhoven, the Netherlands; i Department of Radiology, Maxima Medical Center, De Run 4600, 5504, MB, Veldhoven, the Netherlands; j Department of Radiology, Netherlands Cancer Institute, Plesmanlaan 121, 1066, CX, Amsterdam, the Netherlands; k Department of Radiology, Canisius Wilhelmina Hospital, Weg Door Jonkerbos 100, 6532 SZ, Nijmegen, the Netherlands

**Keywords:** Breast neoplasms, Early detection of cancer, Magnetic resonance imaging, Problem solving

## Abstract

**Purpose:**

Problem solving magnetic resonance imaging (MRI) is used to exclude malignancy in women with equivocal findings on conventional imaging. However, recommendations on its use for women recalled after screening are lacking. This study evaluates the impact of problem solving MRI on diagnostic workup among women recalled from the Dutch screening program, as well as time trends and inter-hospital variation in its use.

**Methods:**

Women who were recalled at screening mammography in the South of the Netherlands (2008–2017) were included. Two-year follow-up data were collected. Diagnostic-workup and accuracy of problem solving MRI were evaluated and time trends and inter-hospital variation in its use were examined.

**Results:**

In the study period 16,175 women were recalled, of whom 906 underwent problem solving MRI. Almost half of the women (45.4%) who underwent problem solving MRI were referred back to the screening program without further workup. The sensitivity, specificity, and positive and negative predictive values of problem solving MRI were 98.2%, 70.0%, 31.1%, and 99.6%, respectively. The percentage of recalled women receiving problem solving MRI fluctuated over time (4.7%–7.2%) and significantly varied among hospitals (2.2%–7.0%).

**Conclusion:**

The use of problem solving MRI may exclude malignancy in recalled women. The use of problem solving MRI varied over time and among hospitals, which indicates the need for guidelines on problem solving MRI.

## Highlights

• Problem solving MRI did correctly refer back women to the screening program.
• The sensitivity and specificity of problem solving MRI were 98.2% and 70.0%.
• Positive and negative predictive values of problem solving MRI were 31.1% and 99.6%.
• By excluding malignancy, problem solving MRI may reduce invasive diagnostic workup.

## List of abbreviations

**Abbreviation Explanation**
BI-RADS: Breast Imaging Reporting and Data System
CESM: Contrast-enhanced spectral mammography
DCIS: Ductal carcinoma in situ
EAPC: Estimated annual percent change
Her2/Neu: Human Epidermal growth factor Receptor 2
LCIS: Lobular carcinoma in situ
MRI: Magnetic resonance imaging
NPV: Negative predictive value
N: Lymph node-negative
N+: Lymph node-positive
PPV: Positive predictive value
ROC: Receiver operating characteristic

## 1 Introduction

Breast cancer is the most commonly diagnosed cancer and a leading cause of cancer death among women, with an estimated 2.1 million new cases and 600,000 deaths in 2018 worldwide [1]. Breast cancer mortality in the Netherlands has declined over the past three decades [2]. This decline has been attributed to early detection of breast cancer through mammography screening and better personalized breast cancer treatment [[3], [4], [5], [6]].

In the Netherlands, biennial screening mammography for breast cancer is offered to women between 50 and 75 years of age. In case of suspicious mammographic findings, women are recalled for additional workup. This workup includes further imaging and, if required, breast biopsies. Various imaging modalities exist, but breast magnetic resonance imaging (MRI) has been recognized as the most sensitive imaging modality for breast cancer detection, with a sensitivity of approximately 90% [[7], [8], [9]]. Breast MRI is most sensitive to vascularized tumors. Indications for breast MRI in patients recalled from screening include problem solving, preoperative planning and monitoring of neoadjuvant chemotherapy [7]. The focus of this article is on breast MRI for problem solving purposes. Problem solving is used to exclude malignancy in women with equivocal findings on conventional imaging and relies on the high negative predictive value (NPV) of breast MRI [7,10].

Very little research has been done regarding the use of breast MRI as a problem solving tool in the setting of an organized screening program. Recent studies have suggested that the NPV of breast MRI is sufficiently high to exclude malignancy, thereby reducing the need to perform invasive diagnostic procedures [11,12]. However, it remains unclear whether breast MRI after screening facilitates the workup of women with inconclusive results by conventional imaging. Breast MRI has been suggested to lead to an unacceptable number of false positive findings that require unnecessary additional examinations and biopsies in the clinical setting, resulting in patient anxiety and increasing healthcare costs [8,9]. As a result, there is no guideline that recommends the use of breast MRI to assess the nature of a lesion when needle biopsy can be performed instead [8].

Despite on-going discussions about appropriate protocols and indications, the use of breast MRI for problem solving purposes is widespread. Over 80% of the European Society of Breast Imaging members reported problem solving MRI as one of the indications for which they used breast MRI [13]. Yet, the impact of problem solving MRI on diagnostic workup in women recalled at screening mammography is largely unknown. Such information is important to develop and improve practice guidelines. Therefore, this study aimed to determine the impact of problem solving MRI on diagnostic workup and to evaluate time trends and inter-hospital variation in its use, using data from women who were recalled at biennial screening mammography in the south of the Netherlands over the past decade.

## 2 Materials and methods

### 2.1 Study population

We retrospectively analyzed women between 50 and 75 years who underwent screening mammography and were recalled in a southern region of the Netherlands between January 1, 2008 and December 31, 2017. Before being screened women were offered the option to opt-out of the use of their data. Two recalled women used this option and were not included. Ethical approval for this study was not necessary according to the Medical Research Involving Human Subjects Act and the study was conducted according to good clinical practice and in accordance with the declaration of Helsinki.

#### 2.1.1 Screening procedure

Details of the biennial screening program have been described previously [[14], [15], [16]]. In short, screening mammograms were read independently by two screening radiologists, who classified the mammograms according to the Breast Imaging Reporting and Data System (BI-RADS) [17,18]. Women with BI-RADS 1 or 2 findings were not recalled and were invited to re-attend the screening program in two years. Women with BI-RADS 0, 4, or 5 findings were recalled for additional workup at a hospital. BI-RADS category 3 was not used in the Dutch screening program, as short-interval follow-up was not available. Initially, when one radiologist classified a mammogram as BI-RADS 1 or 2 and the other radiologist classified it as BI-RADS 0, 4, or 5, the woman in question was recalled without a consensus meeting. From 2015 on, a third radiologist was involved in case of discrepant readings.

### 2.2 Diagnostic workup after recall

Diagnostic workup took place in thirty hospitals. The workup of the majority of women (97.5%, 15,771/16,175) was done in six regional hospitals in the south of the Netherlands. In the hospitals, recalled women received physical examination and underwent additional breast mammographic views. Since 2010 additional tomographic views were also made. The clinical radiologist of the team classified the new imaging findings according to BI-RADS [17,18] and decided whether additional imaging and biopsy procedures were needed to establish a final diagnosis of the mammographic abnormality. If additional workup was needed, breast ultrasounds were usually performed first, according to the indications of the European Society of Breast Imaging [19]. Further workup after breast ultrasound could consist of MRI, percutaneous biopsy, and/or open surgical biopsy, depending on the findings or prior imaging and/or biopsy. The main focus of this study was on breast MRIs performed for problem solving purposes. Problem solving MRIs were performed to determine the nature of lesions seen with conventional imaging. Indications for problem solving MRI included breast asymmetries, masses, and architectural distortions that were not confirmed as benign by conventional assessment because these lesions: 1) could not (easily) be biopsied; 2) could be biopsied but showed discordant results with conventional assessments; or 3) could be biopsied but breast MRI was preferred by the radiologist. For instance, discordant findings could be asymptomatic women with suspicious clinical findings (eg. suspicious palpable abnormality, bloody nipple discharge) but without clear findings at mammography and/or ultrasound. Or discordant findings could include discordant findings between imaging modalities (eg. subtle mammographic findings in which ultrasound findings are negative or may be inconsistent with the mammographic finding). Breast MRI findings were classified according to BI-RADS [17,20]. Women with BI-RADS 4 or 5 lesions after the MRI were routinely biopsied and women with BI-RADS 0 or 3 lesions were either biopsied or followed up, based on the decision of the multidisciplinary breast team. Women with BI-RADS 1 or 2 lesions were referred back to the screening program.

### 2.3 Follow-up of recalled women

Two-year follow-up data of all recalled women was collected. Follow-up data were routinely received by the screening organization, as well as collected by radiologist LEMD and several radiology residents through hospital visits. If a woman was recalled for more than one lesion in a breast or for bilateral lesions, the lesion with the highest BI-RADS classification was considered as the index lesion for recall.

Screen-detected breast cancers were subdivided into ductal carcinoma in situ (DCIS) and invasive cancers. Lobular carcinoma in situ (LCIS) was considered a benign lesion, except for pleomorphic LCIS which was classified as DCIS. Cancers were classified according to the Union for International Cancer Control TNM classification [[21], [22], [23]]. Lymph nodes that contained only isolated tumor cells (<0.2 mm) were considered negative (N-) and lymph nodes that contained micrometastases (0.2–2 mm) or macrometastases (>2 mm) were considered positive (N+). Estrogen and progesterone status were considered positive if ≥ 10% of the cancer cells showed nuclear staining [24]. Human Epidermal growth factor Receptor 2 (Her2/Neu) status was considered positive in case of HER2 3+ or HER2 2+ confirmed with positive in situ hybridization [24].

## 3 Statistical analysis

### 3.1 Primary outcomes

The primary outcome measures of this study were diagnostic workup and diagnostic accuracy of problem solving MRI. Details of workup after problem solving MRI (additional imaging, biopsy and/or follow-up) were collected and displayed in a flowchart. Breast MRI examinations for problem solving purposes involved clinical indications and were considered positive if they showed inconclusive (BI-RADS 0), probably benign (BI-RADS 3), suspicious (BI-RADS 4) or highly suspicious (BI-RADS 5) findings, meaning further examinations were necessary. Breast MRI examinations were considered negative for negative and benign MRI assessments (BI-RADS 1 or 2) as no more workup was needed. The accuracy of breast MRI for the differentiation between benign and malignant lesions was established using receiver operating characteristic (ROC) analysis, with breast MRI BI-RADS findings as the classification variable and final diagnosis (benign versus malignant, based on pathological examination) as the reference variable.

### 3.2 Secondary outcomes

Secondary outcome measures were time trends and inter-hospital variation in the use of problem solving MRI after recall from screening. Joinpoint analyses were performed to assess the significance of changes in time trends. The Joinpoint Regression Program version 4.7 estimated joinpoints at which a significant change in trend occurred [25]. Inter-hospital variation was determined for all hospitals involved in the diagnostic workup. Chi-square tests were performed to statistically compare proportional differences.

For both primary and secondary outcomes, only breast MRIs performed for problem solving within 2 years after screening mammography and before surgery and/or neoadjuvant therapy were included. Women who were referred back to the breast cancer screening program and were later recalled to the hospital for breast MRI because of new breast complaints were excluded from analyses.

All statistical analyses, except for the joinpoint analyses, were performed using IBM SPSS Statistics version 25.0 (IBM SPSS Statistics for Windows, IBM Corp., Armonk, NY). Statistical tests were two-sided and *P*-values less than 0.05 were regarded as statistically significant.

## 4 Results

### 4.1 Workup after problem solving MRI

**Fig. 1:**
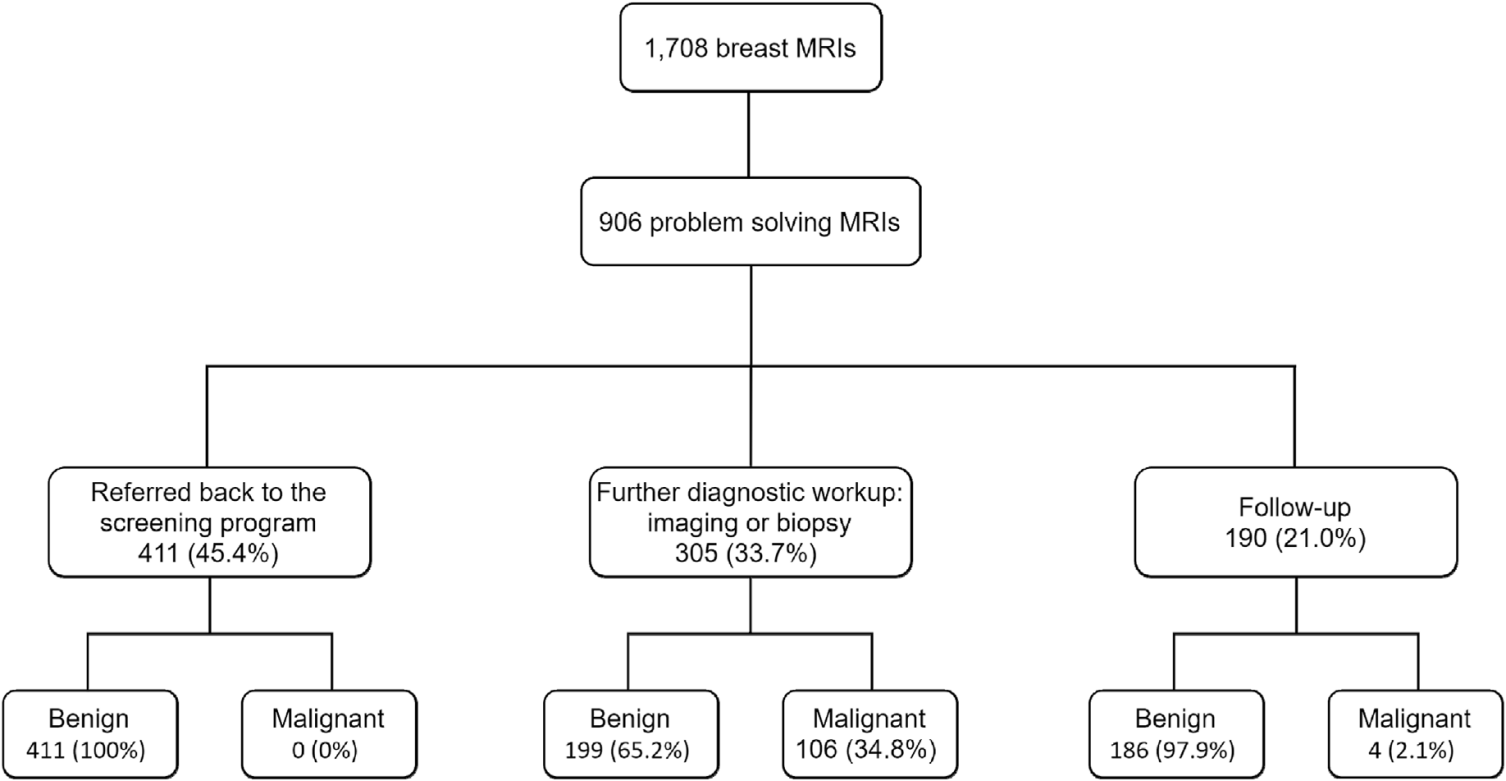
Flowchart with outcomes of breast MRI examinations performed for problem solving in women recalled between 2008 and 2017. MRI, magnetic resonance imaging.

In total, 16,175 women were recalled between January 1, 2008 and December 31, 2017. In these women, 1708 breast MRI examinations were performed of which 906 (53.0%) were for problem solving purposes (Fig. 1). In 786 (46.0%) women breast MRI was performed for preoperative planning and in the remaining 16 women breast MRI was used for other purposes, including surveillance of BI-RADS 3 lesions and screening in women with a family history of breast cancer. Of the 906 women who underwent problem solving MRI, 305 (33.7%) underwent further diagnostic workup. Diagnostic workup was limited to breast imaging in 29 (9.5%) women, whereas 276 (90.5%) women underwent breast biopsy. Of the remaining 601 women, 190 (21.0%) received radiological follow-up. The other 411 (45.4%) women were advised to re-attend the screening program at their next invitation. After a follow-up period of 2 years, final diagnoses were malignant in 110 women and benign in 796 women.

### 4.2 Diagnostic accuracy

**Fig. 2:**
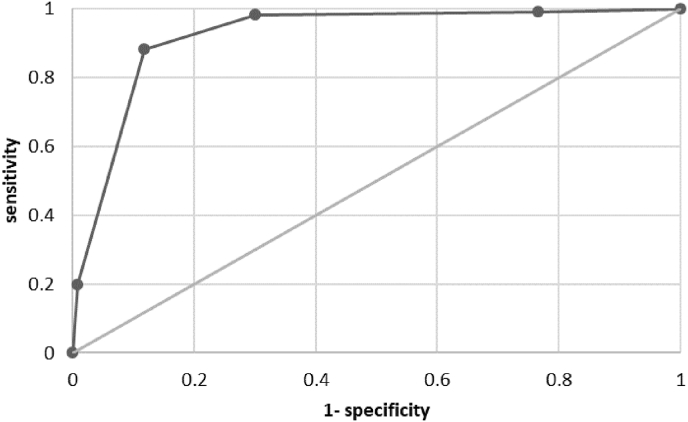
Receiver operating characteristic curve of BI-RADS ratings differentiating between benign and malignant lesions. At a cut-off of >BI-RADS 2 and considering BI-RADS 0 positive, the sensitivity and specificity were 98.2% and 70.0%, respectively. At a cut-off of >BI-RADS 3, the sensitivity and specificity were both 88.2%.

MRI BI-RADS ratings of problem solving examinations were assigned as follows: BI-RADS 0: 7 (1 malignant), BI-RADS 1: 188 (1 malignant), BI-RADS 2: 371 (1 malignant), BI-RADS 3: 149 (10 malignant), BI-RADS 4: 163 (75 malignant), and BI-RADS 5: 28 (22 malignant). ROC analysis revealed an area under the ROC curve of 0.92 (95% CI: 0.90 to 0.95, Fig. 2). Considering BI-RADS 0, 3, 4, and 5 positive and BI-RADS 1 and 2 negative, the sensitivity, specificity, and positive predictive value (PPV) and negative predictive value (NPV) of problem solving MRI were 98.2%, 70.0%, 31.1%, and 99.6%, respectively.

Analyses stratified by age and mammographic abnormalities can be found in Table 1. Women with problem solving MRI were generally younger and the mammographic abnormalities most frequently leading to MRI were asymmetry and architectural distortion. The sensitivity and NPV of problem solving MRI were high in all groups. Specificity did not show any significant differences between the groups, but seems to increase with age. The PPV of problem solving MRI also shows an increasing trend with age, with a statistically significant improvement in PPV for the 60–70 and > 70 group as compared to the <60 group (PPV 20% < 60 vs. 43.6% 60–70 vs. 56.7% > 70). The PPV in women with asymmetries was significantly lower than the PPV in women with a mass. However, it should be noted that the PPV is influenced by the prevalence in the subgroups.

**Table 1:** Diagnostic accuracy of problem solving MRI, stratified by age and mammographic abnormalities.

	% problem solving MRI	Sensitivity (%)	Specificity (%)	PPV (%)	NPV (%)	Prevalence
Age, years						
<60	534/8494 (6.3%)	97.6 (87–100)	67.5 (63–72)	20.0 (18–22)	99.7 (98–100)	41 (7.7%)
60–70	297/5697 (5.2%)	98.1 (90–100)	73.1 (67–79)	43.6 (39–49)	99.4 (96–100)	52 (17.5%)
>70	75/1984 (3.8%)	100 (80–100)	77.6 (65–87)	56.7 (45–68)	100^b^	17 (22.7%)
Mammographic abnormality^a^						
Mass	593/10,110 (5.9%)	100 (95–100)	70.4 (66–74)	32.2 (29–35)	100^b^	73 (12.3%)
Microcalcifications	87/3175 (2.7%)	86.7 (60–98)	65.3 (53–76)	34.2 (26–43)	95.9 (86–99)	15 (17.2%)
Mass with microcalcifications	32/723 (4.4%)	100 (40–100)	57.1 (37–76)	25.0 (18–34)	100^b^	4 (12.5%)
Asymmetry	76/972 (7.8%)	100 (29–100)	82.2 (71–90)	18.8 (12–27)	100^b^	3 (4.0%)
Architectural distortion	102/1096 (9.3%)	100 (77–100)	64.8 (54–75)	31.1 (25–37)	100^b^	14 (13.7%)
Other	16/97 (16.5%)	100 (3–100)	73.3 (45–92)	20.0 (10–37)	100^b^	1 (6.3%)

MRI, magnetic resonance imaging; NPV, negative predictive value; PPV, positive predictive value.

a For 2 women mammographic abnormality was unknown.

b 95% confidence intervals are unknown as zero false negatives occurred in this group.

### 4.3 Breast cancers detected with problem solving MRI

The imaging and histopathological features of malignancies diagnosed in women with problem solving MRI are compared to the features of malignancies diagnosed in women without problem solving MRI in Table 2. Cancers diagnosed in women with problem solving MRI were more often seen as a mass or architectural distortion at screening mammography and less often associated with calcifications (*P* = 0.001). Compared to invasive cancers detected in women without problem solving MRI, invasive cancers diagnosed in women with problem solving MRI were more frequently diagnosed as tumors of the lobular subtype (*P* < 0.001), were generally smaller (*P* < 0.001), more often classified as Bloom & Richardson grade I (*P* = 0.004), and more often estrogen and progesterone receptor-positive (*P* = 0.016 and *P* = 0.015, respectively).

**Table 2:** Imaging and histopathologic features of malignant lesions diagnosed with or without problem solving MRI.

	Tumors detected in women with problem solving MRI (N = 110)	Tumors detected in women without problem solving MRI (N = 3627)	*P* value^a^
Type of cancer			0.171
DCIS	17 (15.5)	755 (20.8)	
Invasive	93 (84.5)	2872 (79.2)	
DCIS grade			0.250
Low	6 (35.3)	144 (19.1)	
Intermediate	5 (29.4)	276 (36.7)	
High	6 (35.3)	333 (44.2)	
Unknown	0	2	
Histology of invasive cancers			<0.001∗
Ductal	62 (66.7)	2282 (79.5)	
Lobular	22 (23.7)	336 (11.7)	
Ductolobular	0	99 (3.4)	
Other	9 (9.7)	155 (5.4)	
Tumor stage of invasive cancers			<0.001∗
T1a + b	54 (59.3)	991 (34.7)	
T1c	20 (22.0)	1302 (45.6)	
T2+	17 (18.7)	562 (19.7)	
Unknown	2	17	
Lymph node status of invasive cancers			0.152
N+	15 (16.7)	645 (23.1)	
N-	75 (83.3)	2146 (76.9)	
Unknown	3	81	
Bloom & Richardson grade			0.004∗
I	55 (60.4)	1247 (43.9)	
II	32 (35.2)	1254 (44.2)	
III	4 (4.4)	338 (11.9)	
Unknown	2	33	
Estrogen receptor status			0.016∗
Positive	88 (97.8)	2577 (90.2)	
Negative	2 (2.2)	280 (9.8)	
Unknown	3	15	
Progesterone receptor status			0.015∗
Positive	75 (83.3)	2040 (71.6)	
Negative	15 (16.7)	808 (28.4)	
Unknown	3	24	
Her2/Neu receptor status			0.211
Positive	5 (5.6)	272 (9.6)	
Negative	84 (94.4)	2575 (90.4)	
Unknown	4	25	
Breast density at screening mammogram			
0–25%	33 (39.3)	913 (31.3)	0.178
25–50%	28 (33.3)	1326 (45.4)	
50–75%	21 (25.0)	615 (21.1)	
75–100%	2 (2.4)	67 (2.3)	
Unknown	26	706	
Mammographic abnormality			0.001∗
Mass	73 (66.4)	2075 (57.2)	
Microcalcifications	15 (13.6)	907 (25.0)	
Mass with microcalcifications	4 (3.6)	334 (9.2)	
Asymmetry	3 (2.7)	55 (1.5)	
Architectural distortion	14 (12.7)	218 (6.0)	
Other	1 (0.9)	38 (1.0)	

DCIS, ductal carcinoma in situ; Her2/Neu, Human Epidermal growth factor Receptor 2; MRI, magnetic resonance imaging; N+, lymph node-positive; N-, lymph node-negative. Values in parentheses are percentages and do not include missing cases. ∗denote statistical significance at *P* < 0.05.

a Chi-square test, missing values were not included in the chi-square tests.

#### 4.3.1 Time trends and hospital variation

**Fig. 3:**
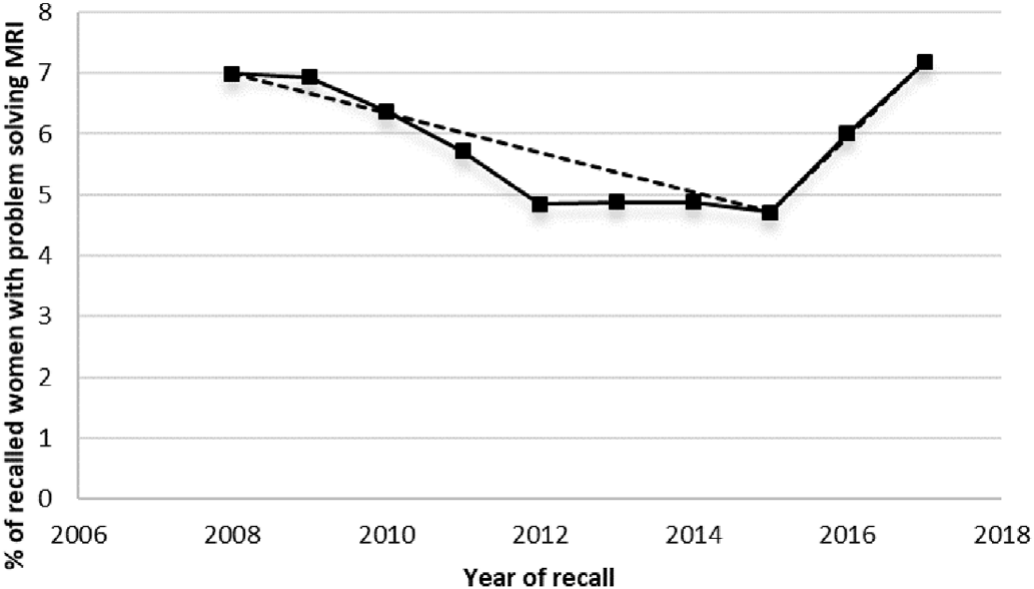
Trends in the use of problem solving MRI after recall between 2008 and 2017. The fitted dashed line was obtained from joinpoint regression analyses. MRI, magnetic resonance imaging.

Rates of problem solving MRI did not significantly differ between women recalled in 2008 and women recalled in 2017 (P = 0.88). However, a statistically significant change in trend occurred in 2015 (Fig. 3, Supplementary Table 1). From 2008 to 2015 problem solving MRI use significantly decreased from 7.0% to 4.7% (P = 0.044), with an estimated annual percent change (EAPC) of −6.0% (95% CI: 8.9 to −3.1), whereas from 2015 to 2017 breast MRI use significantly increased from 4.7% to 7.2% (P = 0.003) with an EAPC of 27.8% (95% CI: 6.1 to 53.9).

**Fig. 4:**
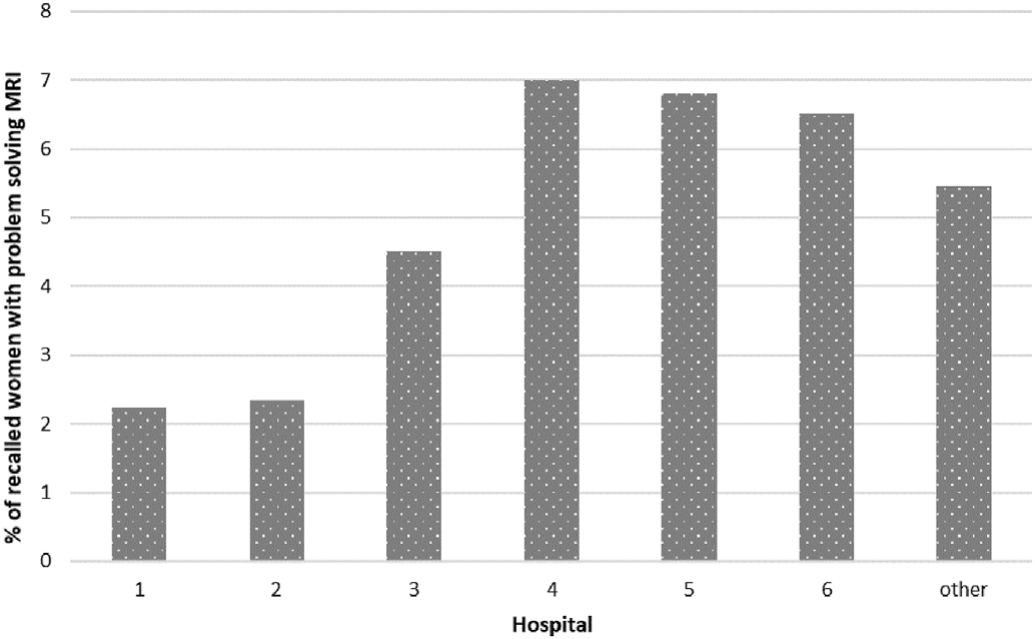
Inter-hospital variation in the use of problem solving MRI for recalled women in the period 2008–2017. Use of breast MRI is shown for the six main hospitals. The ‘other’ category consists of all the remaining hospitals. MRI, magnetic resonance imaging.

The proportion of recalled women who received problem solving MRI significantly varied among hospitals (P < 0.001) and ranged from 2.2% to 7.0% (Fig. 4).

## 5 Discussion

This retrospective 10-year analysis of workup in women recalled at biennial screening mammography found that problem solving MRI reliably excluded malignancy in almost half of the recalled women. This indicates that problem solving MRI may reduce unnecessary diagnostic workup. Furthermore, we found statistically significant variations in the use of problem solving MRI over time and among hospitals.

To our knowledge, this is the first study investigating diagnostic workup of problem solving MRI for women recalled at screening mammography. Based on our results, problem solving MRI reliably excluded malignancy in most recalled women. More than half (51.6%, 411 of 796) of the women with a benign lesion were immediately referred back to the screening program. On the other hand, breast cancer was diagnosed in 110 women, of whom none was falsely referred back to screening. Compared to invasive cancers diagnosed in women without problem solving MRI, invasive cancers in women who underwent problem solving MRI were more often characterized as small, low grade cancers, commonly lobular, and seen as a discrete mass or subtle architectural distortion on mammography (Table 2), features that may be difficult to assess with conventional imaging. These findings underline that problem solving MRI may be useful for further diagnostic workup in situations where biopsy is not (easily) performed and a final diagnosis is not obtained. It should be noted that 385 women with a benign lesion still underwent unnecessary additional examinations and biopsies after problem solving MRI (Fig. 1). Strict selection criteria are thus needed.

Previously published studies have not examined diagnostic workup, but have examined diagnostic accuracy of problem solving MRI in different populations [26,27]. One recent meta-analysis on problem solving MRI concluded that problem solving MRI demonstrates an excellent performance with a pooled sensitivity, specificity, PPV, and NPV of 99%, 89%, 56%, and 100%, respectively [26]. Another meta-analysis showed that problem solving MRI is recommended for diagnosis of malignancy in BI-RADS 4 mammographic calcifications, with a pooled sensitivity and specificity of 92% and 82%, respectively [27]. When considering BI-RADS 0, 3, 4, and 5 positive and BI-RADS 1 and 2 negative, the sensitivity, specificity, PPV and NPV in our study were 98.2%, 70.0%, 31.1%, and 99.6%, respectively. The specificity and with that the PPV are somewhat lower than the pooled specificity and PPV of the meta-analyses. This is likely due to the fact that the women in our study were all already recalled and hence any abnormality on the MRI would lead to biopsy. Nevertheless, sensitivity and NPV were high for all age and mammographic abnormality groups, indicating little false negative findings and the potential to reliably exclude malignancy. Based on our subgroup analyses (Table 1), we may argue that problem solving MRI has the most potential in women aged 60 years or older. As false positive findings are still reported, more research is needed to define strict selection criteria.

Although the available studies on problem solving MRI shed some light on its value, opinions remain divided. There are no guidelines that recommend the use of problem solving MRI when needle biopsies can be performed, even though meta-analyses show that biopsies may be prevented in lesions classified as certainly benign on MRI [8,26,27]. Even the definition of problem solving MRI is not uniform in literature, thereby creating a wide heterogeneity in the available evidence. Studies on breast MRI, performed before 2008, reported rather low pooled diagnostic estimates [28,29] and were quoted by the European Society of Breast Cancer Specialists recommendations as the reason why a negative MRI does not exclude breast cancer [30]. The declining rates in the use of problem solving MRI in the present study may be related to these recommendations and are possibly also a reflection of the lack of national guidelines on problem solving MRI. It is unclear why the use of problem solving MRI increased from 2015 onwards, but improvements in MRI technique and interpretation ability over time and increased diagnostic performance of breast MRI in more recent studies [26,27] may have played a role. In our study we also found substantial variation in the use of problem solving MRI between hospitals, which can only be explained by local preferences of radiologists and physicians. More research is required to answer the question what makes health care professionals decide to use breast MRI for problem solving purposes. Countries have different healthcare reimbursement policies, which may influence the decision for a problem solving MRI. Eventually, local variations in resources (manpower, cost and availability of MRI) and the accessibility to lesions for biopsy are decisive elements in applying or refraining from MRI as problem solving tool. The DENSE trial has already shown that the use of MRI screening in women with extremely dense breast tissue and normal results on mammography resulted in the diagnosis of fewer interval cancers than mammography alone [31]. Before we can make explicit recommendations on the use of MRI for problem solving, in recalled women particularly, cost-effectiveness analysis of this approach is needed. Other imaging modalities, such as digital breast tomosynthesis, targeted and/or whole ultrasound or contrast-enhanced spectral mammography, may eventually also change the value of MRI for problem solving. However, direct comparisons of the different imaging modalities for problem solving are lacking.

Our study has several strengths and limitations. The large study population of 16,175 recalled women with virtually complete 2-year follow-up enabled us to investigate the impact and use of problem solving MRI. Moreover, the study population exclusively consisted of women who were recalled at screening mammography, and to our knowledge, this is the first study to quantify the use of problem solving MRI in a screened population. Abnormalities in women recalled at screening are usually more subtle than those of clinical patients, and therefore MRI presents a greater diagnostic challenge in women recalled at screening.

However, extrapolation of our findings to symptomatic women as well as to other screened populations may be limited. The Dutch screening program differs from screening programs in other countries in several aspects including screening interval, age of screened women, reading strategies, and recall rate. Also, we were not able to retrieve the indications used for problem solving MRI and whether these were adequate or not. Future research needs to more accurately define the indications for problem solving MRI.

In conclusion, we found that breast MRI for problem solving purposes may exclude malignancy in women recalled at screening mammography. This indicates that problem solving MRI may be able to reduce the need for further, often invasive, diagnostic workup. Furthermore, the use of problem solving MRI fluctuated over time and differed between hospitals. These observations demonstrate the importance of more research and consequently evidence-based problem solving MRI guidelines. Eventually, problem solving MRI guidelines and selection criteria need to be better defined and cost effectiveness needs to be elucidated to ensure that breast MRI is used in women who will benefit the most.

## Funding

This research did not receive any specific grant from funding agencies in the public, commercial, or not-for-profit sectors.

## Ethics approval

Women included in this study were not subjected to additional procedures and were not required to follow additional rules of behavior. Hence this study was not subjected to the Medical Research Involving Human Subjects Act and does not warrant ethical approval by an accredited Medical Research Ethics Committee. Furthermore, this study was conducted anonymously and conforms to the principles of the Helsinki Declaration.

## Consent to participate

All women except two (who were excluded) gave consent to participate in this study.

## Consent for publication

Consent for publication was not required because this manuscript does not contain individual person's data.

## Data and/or code availability

The datasets generated during and/or analyzed during the current study are available from the corresponding author on reasonable request.

## Authors’ contributions

Lucien EM Duijm designed the study and maintained the database. Material preparation and data analysis were performed by Jessie JJ Gommers and Lucien EM Duijm. The first draft of the manuscript was written by Jessie JJ Gommers and all authors helped with the interpretation of the results and commented on previous versions of the manuscript. All authors approved the final manuscript.

## Declaration of competing interest

The authors declare that they have no conflict of interest.
