# Supplementary material for: Breast magnetic resonance imaging as a problem solving tool in women recalled at biennial screening mammography: A population-based study in the Netherlands
Source: Breast. 2021 Nov 20;60:279–86. doi: 10.1016/j.breast.2021.11.014 (PMC8628012; doi:10.1016/j.breast.2021.11.014)
Supplement: Multimedia component 1 [file mmc1.doc]

**Breast magnetic resonance imaging as a problem solving tool in women recalled at biennial screening mammography: a population-based study in the Netherlands**

The Breast

Jessie JJ Gommers^*,a^, Adri C Voogd^b^, Mireille JM Broeders^c,d^, Vivian van Breest Smallenburg^e^, Luc JA Strobbe^f^, Astrid B Donkers - van Rossum^g^, Hermen C van Beek^h^, Ritse M Mann^a,i^ and Lucien EM Duijm^j^

*^a^Department of Medical Imaging, Radboud University Medical Center, PO Box 9101, Nijmegen 6500 HB, The Netherlands; ^b^Department of Epidemiology, Maastricht University Medical Center, PO Box 616, Maastricht 6200 MD, The Netherlands; ^c^Department for Health Evidence, Radboud University Medical Center, PO Box 9101, Nijmegen 6500 HB, The Netherlands; ^d^Dutch Expert Center for Screening, PO Box 6873, Nijmegen 6503 GJ, The Netherlands; ^e^Department of Radiology, Jeroen Bosch Hospital, PO Box 90153, ‘s-Hertogenbosch 5200 ME, The Netherlands; ^f^Department of Surgical Oncology, Canisius Wilhelmina Hospital, PO Box 9015, Nijmegen 6500 GS, The Netherlands; ^g^Department of Radiology, Catharina Hospital, PO Box 1350, Eindhoven 5602 ZA, The Netherlands; ^h^Department of Radiology, Maxima Medical Center, PO Box 7777, 5500 MB Veldhoven, The Netherlands; ^i^Department of Radiology, Netherlands Cancer Institute, PO Box 90203, 1006 BE Amsterdam, The Netherlands; ^j^Department of Radiology, Canisius Wilhelmina Hospital, PO Box 9015, 6500 GS Nijmegen, The Netherlands

* Correspondence: Jessie Joke José Gommers; Postal address; Geert Grooteplein 10, Nijmegen 6525 GA, The Netherlands; E-mail: Jessie.Gommers@radboudumc.nl; ORCID identifier: https://orcid.org/0000-0001-6668-7819*

**Supplementary Table 1**–Trends of problem solving MRI use in recalled women by joinpoint analysis, 2008-2017

|  | **Joinpoint analysis** | |  | |
| --- | --- | --- | --- | --- |
|  | **Trend 1** | | **Trend 2** | |
|  | **Period** | **EAPC (95% CI)** | **Period** | **EAPC (95% CI)** |
| **Problem solving MRI** | 2008 - 2015 | -6.0* [-8.9; -3.1] | 2015 - 2017 | 27.8* [6.1; 53.9] |

CI, confidence interval; EAPC, estimated annual percent change; MRI, magnetic resonance imaging. * EAPC is significantly different from 0 at alpha=0.05.

In Supplementary Table 1 the use of problem solving MRI in women recalled after screening is shown for the period 2008-2017. A statistically significant change in trend occurred in 2015. From 2008 to 2015 problem solving MRI use significantly decreased, whereas from 2015 to 2017 breast MRI use significantly increased.
